# Flash Lamp Sintering and Optoelectronic Performance of Silver Nanowire Transparent Conductive Films

**DOI:** 10.3390/ma18235456

**Published:** 2025-12-03

**Authors:** Jiaqi Shan, Ye Hong, Kaixuan Cui, Yifan Xiao, Xingzhong Guo

**Affiliations:** 1 State Key Laboratory of Silicon and Advanced Semiconductor Materials, School of Materials Science and Engineering, Zhejiang University, Hangzhou 310058, China; 21626008@zju.edu.cn (J.S.); 22326095@zju.edu.cn (K.C.);; 2 ZJU-Hangzhou Global Scientific and Technological Innovation Center, Hangzhou 311200, China; 3 Zhejiang X-Way Nano Technology Co., Ltd., Hangzhou 311200, China

**Keywords:** silver nanowire, transparent conductive films, flash lamp sintering, process optimization, sintering mechanism

## Abstract

**Highlights:**

1. Single-pulse flash lamp sintering was employed to achieve millisecond-level rapid sintering and enhance the quality of silver nanowire transparent conductive films (AgNW TCFs).
2. The influence mechanism of the inherent properties of AgNW TCFs, such as sheet resistance and nanowire diameter, on flash lamp sintering was explored and elucidated.
3. The morphological changes of AgNWs during the sintering process were characterized and discussed, revealing a potential intrinsic link between these changes and their crystal structure transformation.

**Abstract:**

Silver nanowire transparent conductive films (AgNW TCFs), as a promising new generation of transparent electrode materials poised to replace ITO, have long been plagued by inadequate optoelectronic performance. Herein, flash lamp sintering was used to facilitate rapid welding of TCFs, and the effects of process parameters and TCFs’ characteristics on the sintering outcomes were investigated. The leveraging of millisecond-scale intense light pulses of flash lamp sintering can achieve the rapid welding of AgNWs, thereby enhancing the optoelectronic performance of TCFs. The TCFs fabricated from 30 nm diameter AgNWs with an initial sheet resistance of 111 Ω/sq exhibited a reduced sheet resistance of 57 Ω/sq post-sintering, while maintaining a transmittance of 93.3%. The quality factor increased from 4.56 × 10^−3^ to 9.09 × 10^−3^ Ω^−1^, and the surface roughness decreased from 6.12 to 5.19 nm after sintering. This work holds significant promise for advancing the continuous production of AgNW TCFs using flash lamp sintering technology, potentially paving the way for high-quality, low-cost, and rapid manufacturing of AgNW TCFs.

## 1. Introduction

Transparent conductive films (TCFs), renowned for their high conductivity and excellent optical transparency, are extensively utilized as transparent electrodes in various fields, including touch screens [1,2], electroluminescent displays (ACEL/OLED) [3,4], solar cells [5,6], and transparent heaters [7,8]. They have consistently been a focal point of research in the field of optoelectronic materials. The materials employed in the fabrication of TCFs primarily encompass indium tin oxide (ITO) [9], carbon nanotubes [10], graphene [11], conductive polymers [12,13], and metal nanowires [14,15]. Among these, ITO stands out and has achieved large-scale production and application due to its mature fabrication process and stable performance, thereby becoming the most prevalent transparent conductive material in use today. Nevertheless, ITO encounters issues such as brittleness, high raw material costs, and limited indium reserves [16]. Notably, with the thriving advancement of flexible electronics in recent years, the field of optoelectronic materials has imposed stricter demands on the flexibility of transparent electrodes, while brittle ITO cannot fulfill the flexibility requirement. Conductive films composed of silver nanowires (AgNWs) exhibit superior flexibility, higher visible light transmittance, and reduced manufacturing costs when compared to ITO films, rendering them a highly regarded alternative to ITO [17].

The preparation principle of AgNW TCFs entails utilizing suitable coating techniques to coat highly conductive AgNWs onto plastic film substrates that exhibit high visible light transmittance. During the coating process, the AgNWs intertwine and arrange themselves to form a comprehensive transparent conductive network. It is evident from this preparation principle that the tightness and robustness of the connections between AgNWs directly influence the conductivity and flexibility of the entire TCFs [18]. Consequently, achieving nanowelding between AgNWs has consistently been a focal point of research in this domain. Currently, the reported AgNW welding technologies primarily encompass chemical welding [19,20], thermal welding [21], joule heating welding [22], hot-press welding [23], plasma pulse welding [24], UV sintering [25], flash lamp sintering [26], etc. Among these techniques, ordinary thermal welding and joule heating welding are straightforward to operate, while they lack self-limiting capabilities and are susceptible to overburning, which can compromise the AgNW conductive network and the substrate [27]. Furthermore, their extended welding duration is incompatible with the roll-to-roll continuous manufacturing process of TCFs. The welding effect of hot-press welding is insignificant, and it often leads to deformation of the TCFs [28]. The technology of plasma pulse welding is still in its nascent stage [24]. Chemical welding is straightforward to perform and yields remarkable welding effects, accounting for its extensive research and reporting [29]. Nevertheless, it may not align with the existing continuous coating technology for AgNW TCFs. Presently, slot-die coating is widely acknowledged as the industry benchmark for roll-to-roll continuous production of AgNW TCFs. To achieve uniform, intact, and consistent TCFs, AgNWs are blended with solvents, film-forming agents, and binders to formulate inks [30]. Upon solvent evaporation, the film-forming agents and binders facilitate the formation of a uniform conductive network by the AgNWs, ensuring robust adhesion to the substrate and offering protection to the nanowires. The presence of these agents and binders diminishes the effectiveness of chemical welding on the shielded AgNWs, rendering it ineffective for welding and introducing additional impurities [31]. Both UV sintering and flash lamp sintering belong to the category of photon sintering, which boasts superior penetration capabilities, enabling it to somewhat counteract the impact of these agents [32]. UV sintering exhibits satisfactory welding results on silver nanowires, but is plagued by relatively prolonged sintering durations, potentially impeding the production efficiency of silver nanowire conductive films [25].

Flash lamp sintering employs wide-spectrum xenon flash lamps to induce photothermal heating at the junctions of AgNWs, facilitating atomic mass transfer between adjacent materials and achieving full welding of the AgNWs [33]. Flash lamp sintering boasts numerous advantages, such as self-limiting characteristics, low cost, high output efficiency, rapid processing capabilities, large-area scalability, and compatibility with roll-to-roll manufacturing processes. Consequently, there have been numerous reports on flash lamp sintering of AgNW TCFs [34]. However, existing research primarily focuses on the principles and self-limiting property of xenon lamp sintering, often examining only a single type of conductive film [35]. In practical applications, flash lamp sintering may encounter different conductive films, with considerable variations in sintering processes for different films. Clarifying the matching mechanism between AgNW TCFs and flash lamp sintering processes is pivotal for advancing the application of flash lamp sintering in the production of AgNW TCFs.

In this study, we employed slot-die coating, which facilitates roll-to-roll preparation, to fabricate AgNW TCFs with diverse sheet resistances using AgNWs with different diameters, based on our previous research [36]. Subsequently, flash lamp sintering was attempted to weld these films, and the sintering process parameters of flash lamp sintering and characteristics of AgNW TCFs were optimized, and the underlying mechanism of flash lamp sintering for achieving AgNW welding was refined.

## 2. Materials and Methods

### 2.1. Flash Lamp Sintering Process

The preparation process of AgNW TCFs is detailed in our previous research [36]. Place the AgNW TCFs on the sample stage surface of the Super Energy Photon Sintering System (produced by FPET Technology Co., Ltd., Ningbo, China). Raise the sample stage to the set position (20 mm from the xenon lamp) and close the chamber door. Set the voltage, pulse length, and number of pulses for flash lamp sintering (this study used a single pulse mode) and then initiate the sintering process. After the pulse ends, lower the sample stage, open the chamber door, and retrieve the samples. The digital image and emission spectrum of the Super Energy Photon Sintering System are shown in Figures S1 and S2.

### 2.2. Characterization

The microstructures of the AgNW TCFs were observed by a scanning electron microscope (SEM, Hitachi Corp., Tokyo, Japan, SU8010). The diameter of the AgNWs was determined using a transmission electron microscope (TEM, Thermo Fisher Scientific, Waltham, MA, USA, Talos F200X S). The UV–visible transmittance spectra of AgNW TCFs were characterized with a UV–visible spectrophotometer (UV-vis, Shimadzu Corp., Kyoto, Japan, UV-3600i Plus). The sheet resistance of AgNW TCFs was measured with a four-point probe sheet resistance meter (Guangzhou four-point probe technology, Guangzhou, China, RTS-9, Φ0.5 mm tips). The visible light transmittance (at 550 nm) of AgNWs was determined with a transmittance/haze tester (Shanghai Yidian Physical Optics Instrument Co., Ltd., Shanghai, China, WGT-S). The surface roughness of the AgNW TCFs was characterized by atomic force microscopy (AFM, Bruker Corporation, Billerica, MA, USA, Dimension ICON).

## 3. Results and Discussion

### 3.1. Optimization of Flash Lamp Sintering Parameters

**Figure 1:**
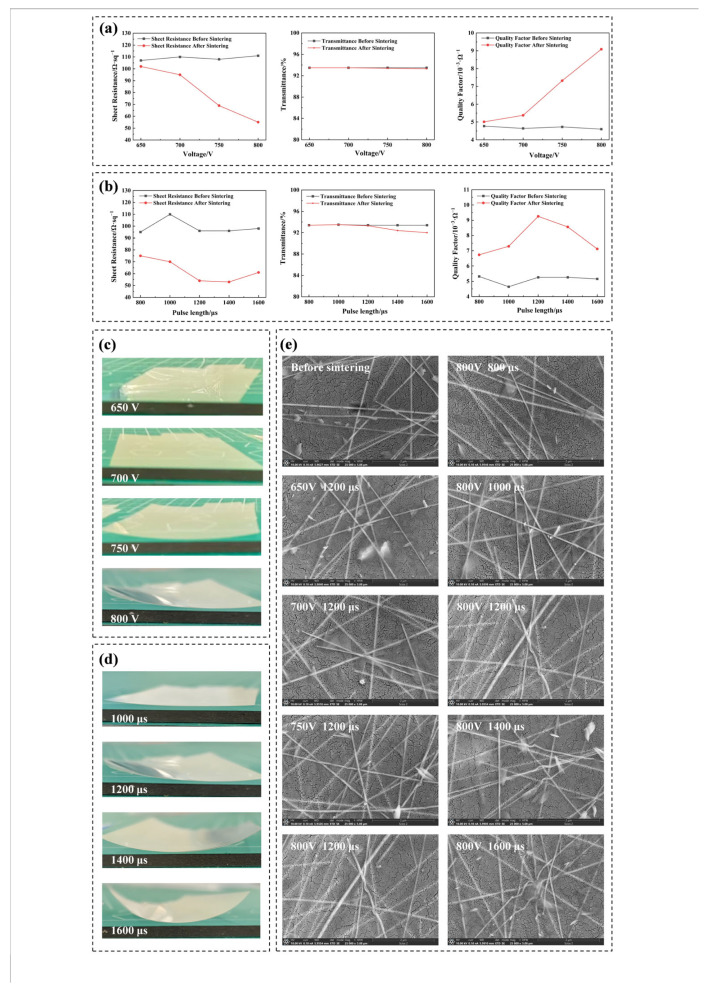
(**a**) Sheet resistance, transmittance, and quality factor of AgNW TCFs before and after flash lamp sintering at various voltages. (**b**) Sheet resistance, transmittance, and quality factor of AgNW TCFs before and after flash lamp sintering at various pulse lengths. (**c**) Digital images of AgNW TCFs after flash lamp sintering at various voltages. (**d**) Digital images of AgNW TCFs after flash lamp sintering at various pulse lengths. (**e**) SEM images (Magnification 25000) of AgNW TCFs prior to flash lamp sintering and subsequent to sintering through various sintering processes.

In the flash lamp sintering process, voltage and pulse length serve as the two pivotal parameters governing the energy and power characteristics of light pulses. In this work, various voltages and pulse lengths are employed for the flash lamp sintering, and the sheet resistance, transmittance, and quality factor of AgNW TCFs before and after sintering are depicted in Figure 1a,b. All transmittance data presented in this study incorporate the PET substrate. When assessing the visible light transmittance of TCFs, a transmittance/haze meter is utilized in this study, instead of UV–visible spectroscopy. The rationale behind this choice is that UV–visible spectroscopy is limited to measuring the transmittance at a specific point on the film, whereas the transmittance/haze meter offers the capability to measure transmittance across a defined area on the film, thereby providing a more comprehensive reflection of the optical properties. The testing principle of the transmittance/haze meter for measuring visible light transmittance is elaborated in the notes beneath Figure S3. Furthermore, to more accurately reflect the enhancement of flash lamp sintering on the photoelectric properties of AgNW TCFs, this study introduces the quality factor as a comprehensive evaluation metric encompassing both the sheet resistance and visible light transmittance of AgNW TCFs. Presently, the formula for calculating the quality factor, which is widely acknowledged, was put forth by G. Haacke [37]. The specific formula is as follows:

(1) $$Q=\frac{T^{10}}{R_{s}}$$

where T is the optical transmittance of the film at a specific wavelength (typically 550 nm), R_s_ is the sheet resistance of the film, and Q is the quality factor.

Therefore, as illustrated in Figure 1a, as the voltage increases, the sheet resistance of AgNW TCFs exhibits a more pronounced decrease before and after sintering, accompanied by a more significant increase in the corresponding quality factor. It suggests that utilizing a high voltage of 800 V for sintering yields superior sintering outcomes. The flash lamp sintering equipment employed in this study has a theoretical maximum voltage of 900 V. However, operating at full capacity places a considerable strain on the power supply, prompting us to refrain from using the maximum voltage for sintering experiments. Furthermore, as the voltage rises, the transmittance of AgNW TCFs after sintering experiences only a slight decline, exerting a minimal influence on the quality factor of TCFs. In the context of the flash lamp sintering system, the voltage dictates the photon energy density and peak power of the light pulse [38]. A higher voltage results in a light pulse with greater energy and peak power, indicative of a higher sintering temperature. This facilitates an enhanced photothermal reaction rate for AgNWs and their junctions [35], thereby accelerating the migration of silver ions from the nanowire surfaces towards the junctions to form welding. Ultimately, it will lead to a reduction in the sheet resistance of TCFs and an improvement in their quality factor. To further investigate the effect of the voltage of flash lamp sintering, this study conducted comprehensive tests and analyses on the appearance and microstructural characteristics of AgNW TCFs before and after sintering under varying voltages. The outcomes are illustrated in Figure 1c,e. From Figure 1c, it is evident that as the voltage escalates, the degree of deformation in the sintered TCFs intensifies, yet they maintain a relatively flat profile. Notably, after sintering at 800 V, slight warping is observed in the conductive film. This is primarily attributed to the higher peak power during flash lamp sintering at 800 V, leading to an elevated peak sintering temperature for AgNW TCFs, which subsequently induces deformation in the PET substrate. Figure 1e reveals that as the voltage increases, the deformation of AgNWs on the TCFs surface gradually becomes more pronounced. Specifically, after sintering at 800 V, significant deformation is evident in the AgNWs, accompanied by welding between them. Additionally, it is discernible that the sintered AgNWs have transformed from their original rod-like shape to a curved and flattened ribbon-like form. During the sintering process, these morphological alterations in the AgNWs effectively augment the contact area between them, thereby decreasing the contact resistance and potentially expediting the welding process among AgNWs.

In addition to voltage, pulse length is equally a crucial factor influencing the effectiveness of flash lamp sintering. Figure 1b illustrates the variations in sheet resistance, transmittance, and quality factor of AgNW TCFs before and after sintering under varying pulse lengths. As evident from Figure 1b, when the pulse length increases, the magnitude of reduction in sheet resistance of AgNW TCFs initially intensifies and then slightly diminishes. Under pulse lengths of 1200 μs and below, the transmittance of TCFs after sintering remains relatively unchanged. However, a notable decline in transmittance is observed for TCFs sintered under pulse lengths of 1400 and 1600 μs. Consequently, the quality factor of TCFs post-sintering exhibits varying degrees of improvement compared to before sintering, attributed to the decrease in sheet resistance. Concurrently, the quality factor of TCFs after sintering demonstrates a trend of initially increasing and then decreasing with the augmentation of pulse length, suggesting that sintering under a pulse length of 1200 μs yields superior sintering results. In the context of flash lamp sintering systems, pulse length directly impacts the energy distribution duration and peak power of the light pulse [38]. Firstly, as pulse length increases, the prolonged energy distribution duration escalates, the prolonged energy distribution duration of the light pulse allows more time for silver ions on the surface of AgNWs to migrate towards their overlap regions and achieve welding. Simultaneously, the extension of energy distribution duration may lead to a decrease in the peak power of the light pulse, potentially compromising the sintering effectiveness. To further investigate the influence pattern of pulse length on the effect of flash lamp sintering, the appearance and microstructural morphology of AgNW TCFs before and after sintering under varying pulse lengths were investigated, as presented in Figure 1d,e. As observed in Figure 1d, with the increase in pulse length, the degree of deformation in the sintered TCFs intensifies. Notably, when the pulse length reaches 1400 and 1600 μs, significant warping is evident in the sintered TCFs, suggesting that excessively long pulse lengths may lead to heat accumulation on the TCF surface, ultimately causing deformation of the PET substrate. From Figure 1e, it is evident that as pulse length increases, the AgNWs on the TCFs surface undergo notable deformation. Specifically, at a pulse length of 1600 μs, the AgNWs on the TCFs surface exhibit a ‘fusing and breaking’ phenomenon. The extension of pulse length prolongs the sintering duration, allowing the substantial heat generated by the photothermal effect on the AgNWs of the TCFs surface to accumulate, leading to deformation of the AgNWs. When this heat accumulation exceeds a certain threshold, it results in the ‘fusing and breaking’ of the AgNWs. In addition, Figure 1e reveals that the AgNWs on the surface of TCFs co-form a film with cellulose, aligning with our previous research findings [36]. Prior to sintering, the AgNWs in TCFs are predominantly located on the surface of the cellulose. Nevertheless, following sintering, particularly under the conditions of 800 V and 1200 μs, some of these nanowires become embedded within the cellulose film. This embedding could be attributed to the melting of cellulose adjacent to the nanowires due to the heat generated during sintering. Such a phenomenon may positively impact the flatness of AgNW TCFs. As a result, a voltage of 800 V and a pulse length of 1200 μs are the optimal process parameters for flash lamp sintering in this work.

### 3.2. Effect of AgNW TCFs’ Inherent Characteristics for Flash Lamp Sintering

**Figure 2:**
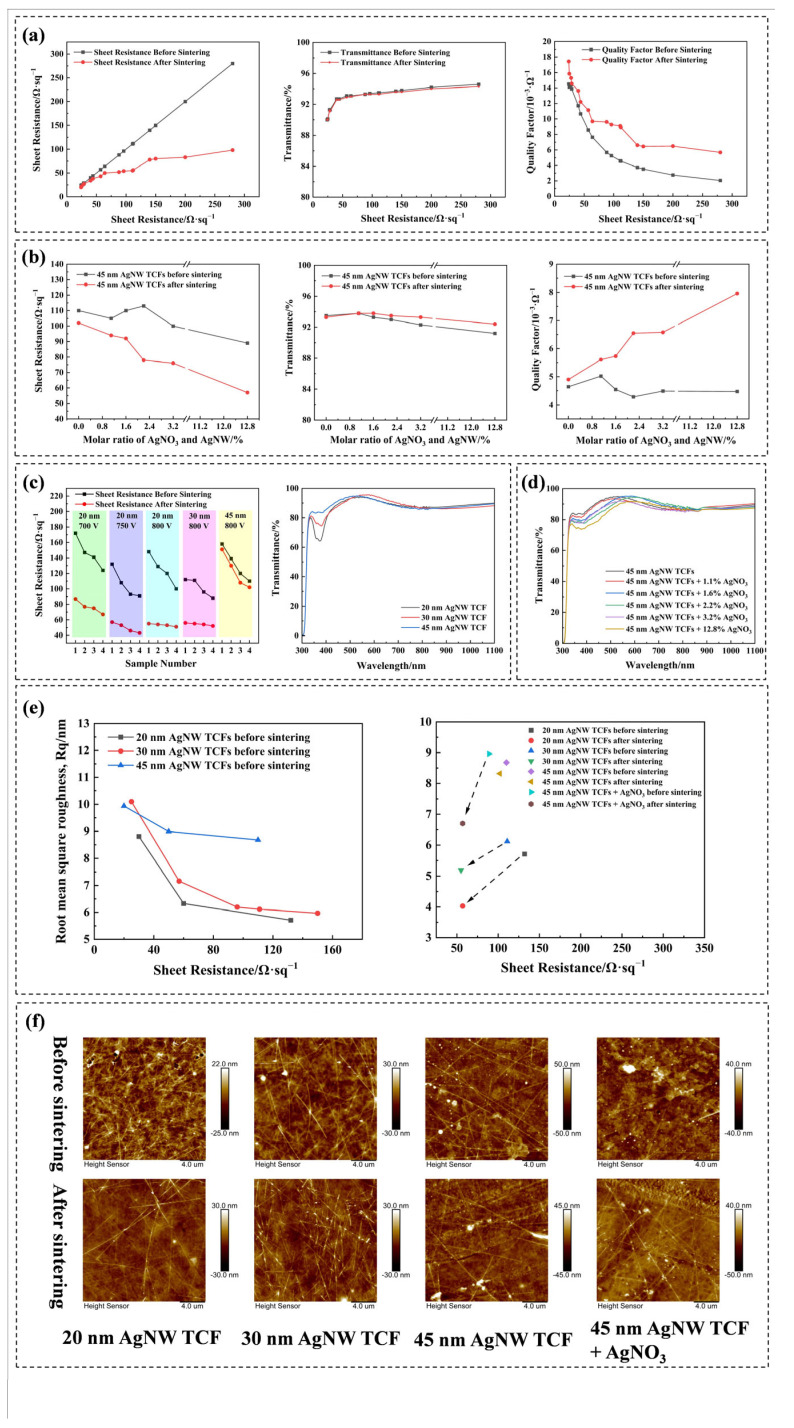
(**a**) Sheet resistance, transmittance, and quality factor of AgNW TCFs, featuring different sheet resistances, before and after sintering. (**b**) Sheet resistance, transmittance, and quality factor of TCFs fabricated from 45 nm AgNWs with varying proportions of AgNO_3_ added, before and after sintering. (**c**) Variations in sheet resistance before and after sintering and UV-vis spectra of TCFs fabricated from AgNWs of varying diameters. (**d**) UV-vis spectra of TCFs fabricated from 45 nm AgNWs with varying proportions of AgNO_3_ added. (**e**) Surface roughness of TCFs fabricated from AgNWs of varying diameters before and after sintering. (**f**) AFM images of AgNW TCFs before and after sintering.

In addition to sintering parameters, the intrinsic properties of the AgNW TCFs, including the sheet resistance of TCFs and the diameter of AgNWs, exert a notable influence on the sintering effectiveness of AgNW TCFs. Figure 2a demonstrates the trends in sheet resistance, transmittance, and quality factor of AgNW TCFs with varying sheet resistances after undergoing flash lamp sintering at 800 V and a pulse length of 1200 μs. As illustrated in Figure 2a, AgNW TCFs with higher sheet resistances exhibit a more pronounced reduction in sheet resistance after sintering, accompanied by a more significant enhancement in the quality factor. Notably, the primary approach adopted in this work to fabricate AgNW TCFs with different sheet resistances involved adjusting the concentration of AgNWs in the conductive ink, an approach grounded in our previous research findings [36]. Consequently, TCFs with lower sheet resistances feature a higher density of AgNWs on their surface, which is consistent with the SEM images of TCFs with varying sheet resistances presented in Figures S4 and S5. The aforementioned observations can be elucidated using 2 formulas, as detailed below:

R_total = R_bulk + R_contact(2)

(3) $$\frac{1}{R\_contact}=\frac{1}{R\_cont1}+\frac{1}{R\_cont2}+\ldots +\frac{1}{R\_contN}$$

where R_total is the resistance of AgNW TCFs, R_bulk is the volumetric resistance of all AgNWs within AgNW TCFs, R_contact is the contact resistance between AgNWs in AgNW TCFs, and R_conN is the contact resistance along a specific path in AgNW TCFs.

According to Ragnar Holm’s classic theory of electrical contacts [39], the resistance of AgNW TCFs comprises the bulk resistance of AgNWs and the contact resistance between them, as illustrated in formula (2). Drawing upon Percolation Theory [40], when current traverses a conductive network formed by AgNWs, multiple parallel pathways exist. The contact resistance along each pathway can be considered as being in series, whereas different pathways are in parallel, as depicted in formula (3). In this research, TCFs exhibiting lower sheet resistance possess a greater number of AgNWs on their surface. Consequently, the conductive network formed by these numerous AgNWs encompasses more conductive pathways. By incorporating formula (3), it becomes evident that an increase in conductive pathways can effectively diminish the contact resistance of TCFs. Hence, for the AgNW TCFs with lower sheet resistance investigated, the proportion of contact resistance within their overall resistance composition is relatively low. The primary mechanism of flash lamp sintering lies in facilitating welding between AgNWs through light pulses, thereby decreasing the contact resistance and ultimately reducing the sheet resistance of TCFs. This elucidates the phenomenon where the sheet resistance of low-resistance TCFs undergoes insignificant reduction following flash lamp sintering.

Meanwhile, as depicted in Figure 2a, TCFs featuring lower sheet resistance demonstrate higher quality factors. This observation may stem not solely from the formula used to calculate the quality factor but also, to a significant extent, from the abundance of conductive pathways on the surface of TCFs with reduced sheet resistance. Although TCFs with lower sheet resistance boast higher quality factors, their light transmittance decreases markedly, particularly when the sheet resistance falls below 50 Ω/sq, at which point a notable decline in light transmittance is evident. More crucially, this study achieved low-sheet-resistance TCFs by adjusting the concentration of AgNWs in the conductive ink, a method that substantially elevates the manufacturing cost of AgNW TCFs. Upon comprehensive consideration of the light transmittance, quality factor, and manufacturing cost of AgNW TCFs, based on the findings presented in Figure 2a, we discovered that TCFs with a sheet resistance of approximately 100 Ω/sq undergo a substantial reduction in sheet resistance (from 111 to 57 Ω/sq) and a notable increase in quality factor (from 4.56 × 10^−3^ to 9.09 × 10^−3^ Ω^−1^) after flash lamp sintering, while maintaining a high level of light transmittance (93.3%) and a relatively low manufacturing cost. Likewise, unsintered AgNW TCFs with a sheet resistance of 57 Ω/sq exhibit a quality factor of 8.58 × 10^−3^ Ω^−1^ and a light transmittance of 93.1%. Evidently, sintered AgNW TCFs exhibit superior quality factors and light transmittance. The manufacturing costs of AgNW TCFs can be compared by examining the areal density of AgNWs on their surfaces. This areal density can be determined based on the volume of conductive ink utilized in slot-die coating and the concentration of AgNWs within that ink [35]. Upon calculation, TCFs with a pre-sintering sheet resistance of 111 Ω/sq exhibited an areal density of AgNWs on their surface of 18.9 mg/m^2^, whereas TCFs with a sheet resistance of 57 Ω/sq demonstrated an areal density of 28.4 mg/m^2^. These findings suggest that flash lamp sintering can decrease the raw material costs associated with the production of AgNW TCFs by approximately 33%.

The effectiveness of flash lamp sintering is influenced not only by the sheet resistance but also by the diameter of the AgNWs utilized in the fabrication of the TCFs. As illustrated in Figure 2c, TCFs composed of 20 nm diameter AgNWs exhibit a notable decrease in sheet resistance after undergoing flash lamp sintering at 700 V. Conversely, TCFs made from 45 nm diameter AgNWs show no significant change in sheet resistance after sintering at 800 V. This observation is further corroborated by the SEM images (Figure S6) of TCFs crafted from AgNWs of varying diameters, both before and after sintering. Specifically, no welding traces were discernible in the TCFs fabricated from 45 nm AgNWs post-sintering, and the AgNWs remained morphologically unaltered. The UV-vis spectra of TCFs made from AgNWs with three distinct diameters, as depicted in Figure 2c, offer insights into the underlying reasons for these observations. The transmittance of TCFs within the 320–500 nm wavelength range diminishes as the diameter of the AgNWs decreases, suggesting that thinner AgNWs demonstrate enhanced absorption of light within this wavelength band, which coincides with the primary energy distribution of the xenon lamp source employed for flash lamp sintering (as depicted in Figure S2). An in-depth analysis of the origin of this phenomenon is related to the principle of flash lamp sintering. The principle of flash lamp sintering primarily relies on the surface plasmon resonance effect and the photothermal effect of the silver nanowires. The smaller the diameter of the silver nanowires, the larger their specific surface area, resulting in a more remarkable surface plasmon resonance effect and a more enhanced photothermal effect.

To further validate our interpretation and enhance the absorption of pulsed light by TCFs fabricated from 45 nm AgNW, we incorporated varying proportions of silver nitrate (AgNO_3_) into the conductive ink utilized in the preparation of TCFs, serving as a sintering aid. The variations in sheet resistance, transmittance, and quality factor of the TCFs, subsequent to sintering at 800 V with AgNO_3_ added, are depicted in Figure 2b. It is evident that the incorporation of AgNO_3_ notably enhances the efficacy of flash lamp sintering on TCFs, resulting in a substantial improvement in the quality factor of the sintered TCFs. Figure 2d presents the UV-vis spectra of TCFs prepared using 45 nm AgNW with different amounts of AgNO_3_ incorporated. It is noted that as the quantity of AgNO_3_ added increases, the absorption of visible light within the wavelength range of 320–500 nm by the resultant TCFs gradually intensifies, suggesting that the addition of AgNO_3_ amplifies the responsiveness of AgNW TCFs to pulsed light utilized in flash lamp sintering. This elucidates the rationale behind the enhanced flash lamp sintering effectiveness of AgNW TCFs upon the addition of AgNO_3_. It is speculated that the underlying mechanism may be related to the surface plasmon resonance effect of nanosilver. The added silver nitrate may precipitate during the drying process of the conductive film, forming nanosilver or silver oxide nanoparticles on the film surface. These small-sized silver nanoparticles can also induce a significant surface plasmon resonance effect, compensating for the insufficient resonance effect of the relatively thick (45 nm) silver nanowires.

Flash lamp sintering not only reduces the sheet resistance of AgNW TCFs but also effectively diminishes their surface roughness. The variation in the root mean square surface roughness (R_q_) of AgNW TCFs before and after sintering is illustrated in Figure 2e, with the corresponding AFM images presented in Figure 2f and Figure S7. It is depicted from Figure 2e that the surface roughness of AgNW TCFs diminishes as their sheet resistance escalates. TCFs fabricated from 45 nm AgNWs exhibit a greater surface roughness owing to their thicker diameters. Moreover, their surface roughness remains largely unchanged after sintering, attributed to their weaker responsiveness to xenon lamp light. Upon the addition of AgNO_3_, the sintering efficacy of TCFs made from 45 nm AgNWs is enhanced, leading to a reduction in sheet resistance from 89 to 57 Ω/sq, accompanied by a decrease in surface roughness from 8.96 to 6.7 nm. Following sintering, TCFs produced from 20 nm AgNWs witness a decline in sheet resistance from 132 to 57 Ω/sq, along with a reduction in surface roughness from 5.71 to 4.03 nm. After sintering, the sheet resistance of TCFs fabricated from 30 nm AgNWs decreased from 111 to 55 Ω/sq, accompanied by a reduction in surface roughness from 6.12 to 5.19 nm. From Figure 2f and Figure S7, the surface irregularities of TCFs are predominantly influenced by the AgNWs, and TCFs with higher sheet resistance possess fewer AgNWs on their surface, consequently exhibiting lower roughness. The AFM images of sintered TCFs reveal a notable decrease in the number of AgNWs, which is likely attributed to the absorption of pulsed light energy by AgNWs on the TCFs’ surface during sintering. It results in heat accumulation and the ‘melting’ of the adjacent cellulose film, thereby allowing the AgNWs to become deeply embedded within the ‘melted’ cellulose.

### 3.3. Effect of AgNW TCFs’ Inherent Characteristics for Flash Lamp Sintering

**Figure 3:**
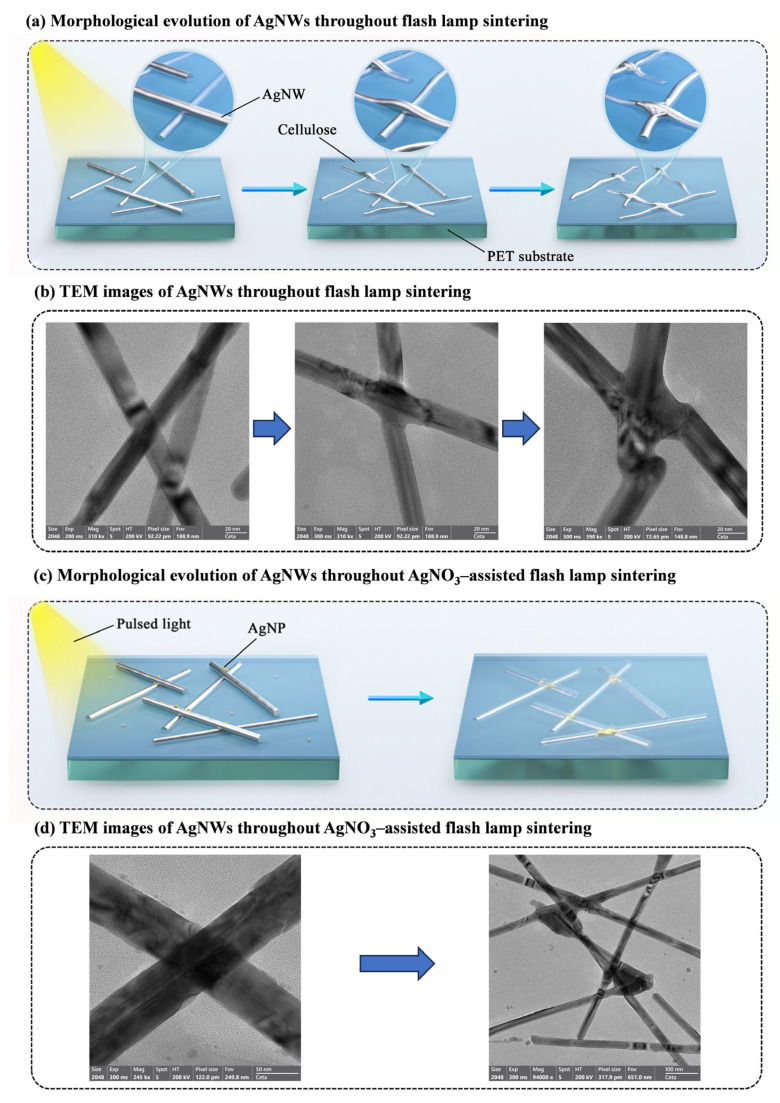
(**a**) Morphological evolution of AgNWs throughout flash lamp sintering. (**b**) TEM images of AgNWs throughout flash lamp sintering. (**c**) Morphological evolution of AgNWs throughout AgNO_3_-assisted flash lamp sintering. (**d**) TEM images of AgNWs throughout AgNO_3_-assisted flash lamp sintering.

As an efficient welding technology for AgNWs, the sintering mechanism of flash lamp sintering has been extensively researched. Drawing upon existing relevant literature reports [32,33,35,41,42,43] and the experimental phenomena observed in this study, we aim to further explore and refine the flash lamp sintering mechanism of AgNW TCFs. Initially, our study revealed that TCFs composed of thinner AgNWs exhibit a superior response to light pulses, facilitating direct welding between AgNWs via flash lamp sintering. The schematic illustration of the morphological evolution of AgNWs during this process is depicted in Figure 3a. Prior to sintering, AgNWs assume a rod-like shape and are merely in contact with one another through overlapping. As flash lamp sintering proceeds, the junctions of AgNWs, serving as the points of maximum plasmon resonance photothermal effect [35], undergo local morphological transformations first, with the AgNWs bending and transitioning from rod-shaped to strip-shaped. As the sintering reaction deepens, similar morphological transformations occur across all sections of the AgNWs, and notable welding phenomena become evident at their junctions. These morphological transformations in AgNWs provide a valid explanation for the experimental observation that both the sheet resistance and surface roughness of TCFs decrease following flash lamp sintering. To more authentically and clearly observe and capture these morphological changes in AgNWs, this study employs a method of ultrasonically dispersing AgNW TCFs in an alcohol/water mixture before and after sintering, and subsequently examines them under TEM. The results are depicted in Figure 3b, which aligns well with the morphological transformation process observed in Figure 3a.

**Figure 4:**
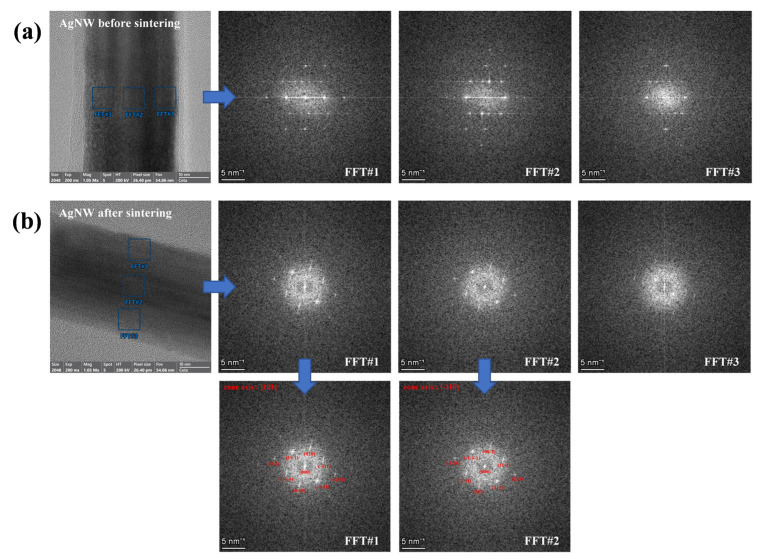
(**a**) Electron diffraction pattern of AgNWs before sintering. (**b**) Electron diffraction pattern of AgNWs after sintering.

Based on the literature reports pertinent to nano-silver catalysis [44,45], we postulate that the morphological transformations observed in AgNWs are all intricately linked to their plasmonic resonance photothermal effect. When subjected to light pulses, AgNWs exhibit a photothermal effect, which triggers the generation of high temperatures and alterations in the electric field on their surfaces. These high temperatures prompt the bending and deformation of AgNWs, whereas the synergistic action of high temperatures and electric field variations accelerates the oxidation of Ag to Ag^+^. Subsequently, some of these Ag^+^ undergo reduction back onto the AgNW surface, contributing to the morphological transition of AgNWs from rod-like to strip-like structures. Conversely, another fraction of Ag^+^ migrates towards the junctions of AgNWs and undergoes reduction at these sites, thereby facilitating the welding between AgNWs. Additionally, this study conducted electron diffraction analysis of AgNWs before and after sintering to validate the mechanism underlying their morphological changes. The results are depicted in Figure 4. Figure 4a presents electron diffraction images captured from three distinct locations on the surface of AgNWs prior to sintering. All three images exhibit diffraction patterns characteristic of a twinning structure, aligning with the literature reports that AgNWs possess a penta-twinned structure [46,47]. The electron diffraction image of sintered AgNWs presented in Figure 4b fails to demonstrate the features of a five-fold twinned crystal. Rather, it reveals two electron diffraction spots that align with the face-centered cubic single-crystal diffraction pattern of Ag. We consulted the mp-124 crystal structure of Ag and employed CrysTBox software (version 1.10) to annotate the crystal zone axes and crystal planes of the single-crystal diffraction spots. Consequently, we postulate that AgNWs with a five-fold twinned structure are more inclined to exhibit a rod-like morphology. Under the influence of the photothermal effect, rod-shaped AgNWs undergo oxidation and reduction processes, leading to structural rearrangement and the formation of partial face-centered cubic single-crystal structures. These face-centered cubic single-crystal AgNWs may be more prone to adopting a strip-like morphology.

Secondly, the TCFs composed of thicker AgNWs in this study demonstrate a weaker response to light pulses, necessitating the incorporation of AgNO_3_ as a sintering aid. The schematic diagram depicting the morphological evolution of AgNWs throughout the AgNO_3_-assisted flash lamp sintering process is shown in Figure 3c. Prior to sintering, the AgNWs retain their rod-like morphology, with the surface of the AgNW TCFs exhibiting a small number of nano silver particles (AgNPs) formed through the reduction of AgNO_3_. As the flash lamp sintering proceeds, the AgNPs on the surface undergo photothermal effects due to the light pulses. These AgNPs are then reduced to Ag^+^ and migrate along the AgNW surface towards the junctions of the AgNWs. A substantial amount of Ag^+^ migrates and reduces at these junctions, leading to the welding of the AgNWs. In this sintering process, which is facilitated by an additional Ag source, a more prominent ‘silver deposition and coating’ phenomenon is observed at the AgNW junctions. As illustrated in Figure 3d, we further validated the occurrence of this phenomenon through TEM observation of the AgNWs before and after sintering. This finding not only elucidates the mechanism by which AgNO_3_ functions as a sintering aid but also corroborates that the migration of Ag^+^ on the AgNW surface serves as the primary cause and driving force for achieving welding between the AgNWs.

## 4. Conclusions

Flash lamp sintering technology was utilized to weld AgNW TCFs for enhancing their optoelectronic properties and surface planarity. A voltage of 800 V and a pulse length of 1200 μs constitute the optimal sintering parameters for flash lamp sintering of AgNW TCFs. The AgNW TCFs with a sheet resistance of approximately 100 Ω/sq are deemed more suitable for flash lamp sintering treatment, and the manufacturing cost of AgNW can be decreased by roughly 33% compared to unsintered TCFs of equivalent sheet resistance. The AgNW TCFs fabricated from finer AgNWs (20 and 30 nm) exhibit a marked enhancement in their quality factor following flash lamp sintering, while AgNWs with 45 nm exhibit a weaker response to pulsed light during sintering. AgNO_3_ as a sintering aid can be reduced to form AgNPs and bolster the responsiveness of TCFs to pulsed light for improving the sintering efficacy of TCFs. During the sintering process, AgNWs undergo plasmon resonance-induced photothermal effects upon exposure to pulsed light, and localized high temperatures and alterations in the electric field on their surfaces lead to bending deformation and the generation of surface Ag^+^. A fraction of these surface Ag^+^ is reduced to facilitate the morphological transition of AgNWs from rod-like to strip-like structures and the crystal transformation of the AgNWs from quintuple twinning to a single crystal, and another portion of Ag^+^ migrates to the junctions of AgNWs to achieve welding.

## Data Availability

The original contributions presented in this study are included in the article/Supplementary Material. Further inquiries can be directed to the corresponding author.

## Supplementary Materials

The following supporting information can be downloaded at: https://www.mdpi.com/article/10.3390/ma18235456/s1, Figure S1: The digital image of the Super Energy Photon Sintering System; Figure S2: The emission spectrum (200–1000 nm) of the Super Energy Photon Sintering System; Figure S3: Digital image of a transmittance/haze tester; Figure S4: SEM images (Magnification 5000) of 30 nm AgNW TCFs with different sheet resistances after sintering; Figure S5: SEM images (magnification 25000) of 30 nm AgNW TCFs with different sheet resistances after sintering; Figure S6: SEM images (magnification 25000) of 20, 30 and 45 nm AgNW TCFs before and after sintering; Figure S7: AFM images of 20, 30 and 45 nm AgNW TCFs with different sheet resistances.
