# Supplementary material for: Flash Lamp Sintering and Optoelectronic Performance of Silver Nanowire Transparent Conductive Films
Source: Materials (Basel). 2025 Dec 3;18(23):5456. doi: 10.3390/ma18235456 (PMC12692753; doi:10.3390/ma18235456)
Supplement: Supplementary file 1 [file materials-18-05456-s001.zip › materials-4011927-supplementary.pdf]

Flash Lamp Sintering and Optoelectronic Performance of Silver Nanowires Transparent Conductive Films

Jiaqi Shan [a,b], Ye Hong [c], Kaixuan Cui [a], Yifan Xiao [a], Xingzhong Guo [a,b].

[a] State Key Laboratory of Silicon and Advanced Semiconductor Materials, School of Materials Science and Engineering, Zhejiang University, Hangzhou 310058, PR China.
[b] ZJU-Hangzhou Global Scientific and Technological Innovation Center, Hangzhou 311200, PR China.
[c] Zhejiang X-Way Nano Technology Co., Ltd., Hangzhou 311200, PR China.

Supporting information

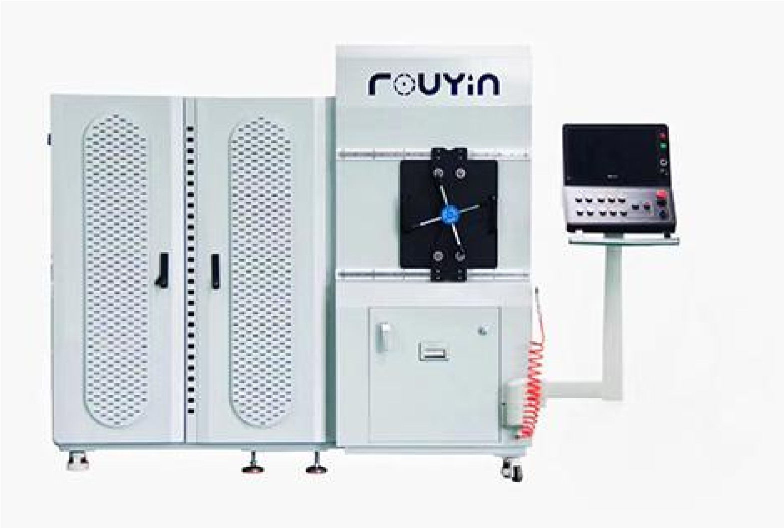

Figure S1. The digital image of the Super Energy Photon Sintering System

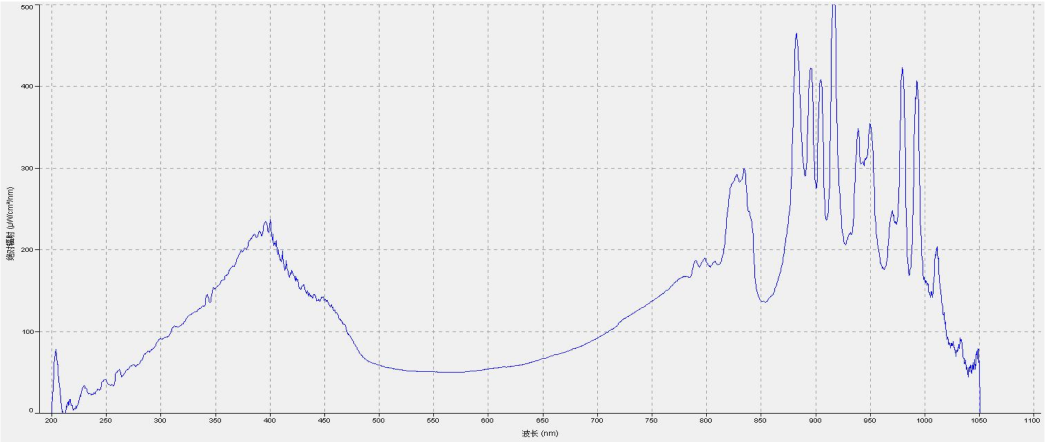

Figure S2. The emission spectrum (200-1000 nm) of the Super Energy photon sintering system (Ps: X axis: wavelength/nm; Y axis: $\mu W \cdot cm^{-2} \cdot nm^{-1}$)

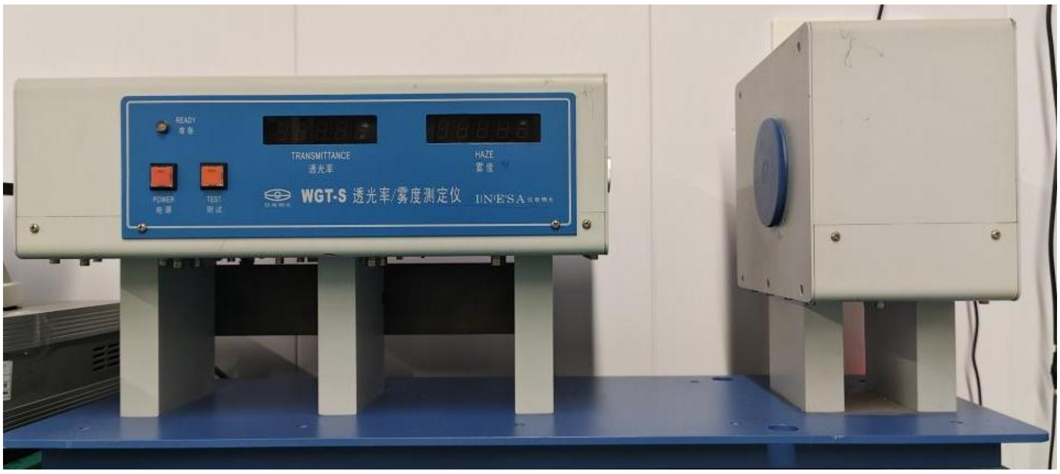

Figure S3. Digital image of a transmittance/haze tester

(Ps: The light source of this device employs a DC 12V 50W halogen tungsten lamp equipped with a color temperature filter, designed to simulate the spectral distribution of sunlight ranging from 380 to 780 nm. The calculation method used by the device to measure transmittance is the ratio of transmitted luminous flux to incident luminous flux: $\mathbf{T = \Phi_t/\Phi_i * 100\%}$.

The device employs an integrating sphere receiving system, and the receiver is equipped with a viewing function correction plate. Therefore, its received luminous flux function is as follows:

$$\boldsymbol{\Phi_\upsilon = K_m \int_{380}^{780} \Phi_e(\lambda) \cdot V(\lambda) d\lambda}$$

V(λ) is a standard visual function, which has the highest weight near 550 nm. Therefore, the transmittance values measured by this device primarily reflect the transmittance of the conductive film near 550 nm. This device adheres to the ASTM D1003 standard, and its test results reflect the actual transparency perceived by the human eye. The visible light transmittance measured by this device for TCFs can better reflect the transmittance characteristics of the entire film, while UV-vis spectroscopy can only measure the spectrum at a single location on the surface of the TCFs. Therefore, in this study, the measurement of visible light transmittance of TCFs is conducted using this device rather than UV-vis spectroscopy. UV-vis spectroscopy is mainly used in this study to analyze the response characteristics of the thin film to light of different wavelengths.)

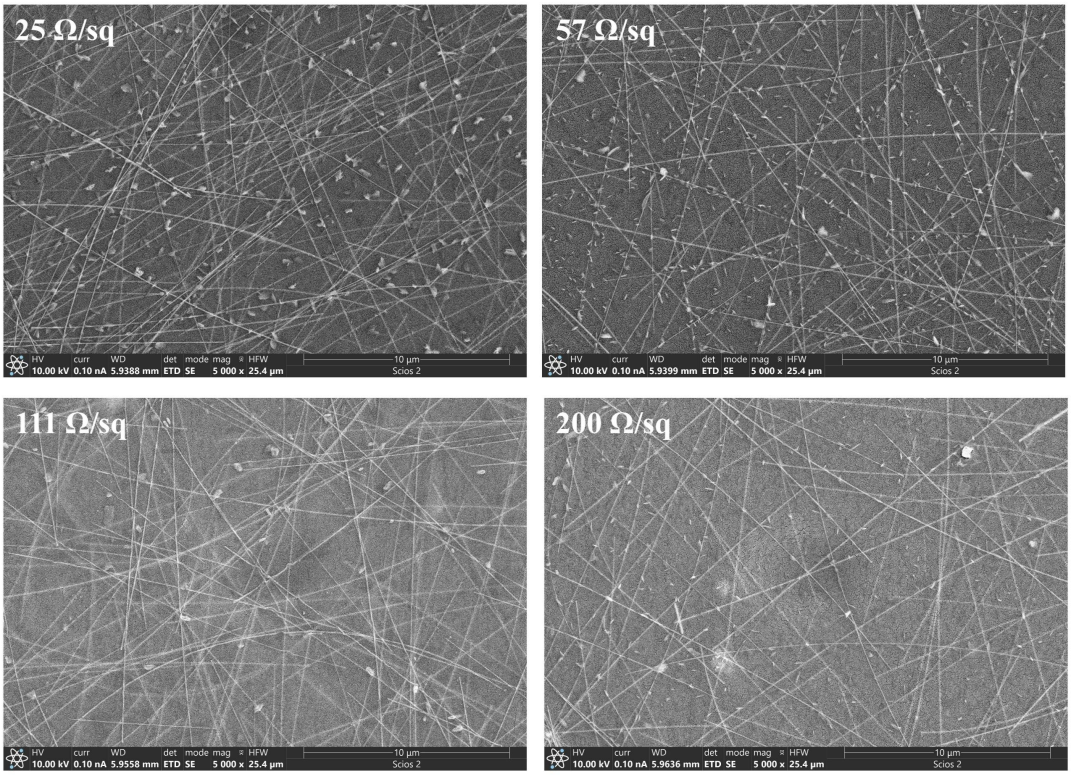

Figure S4. SEM images (Magnification 5000) of 30 nm AgNW TCFs with different sheet resistances after sintering

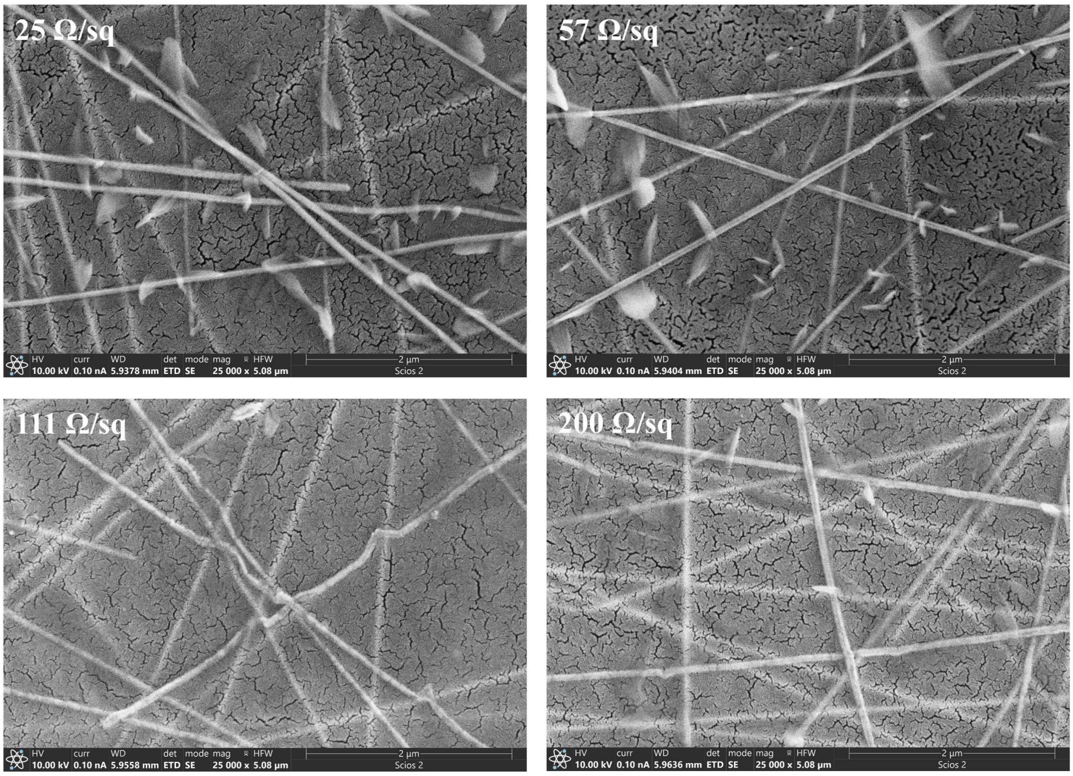

Figure S5. SEM images (Magnification 25000) of 30 nm AgNW TCFs with different sheet resistances after sintering

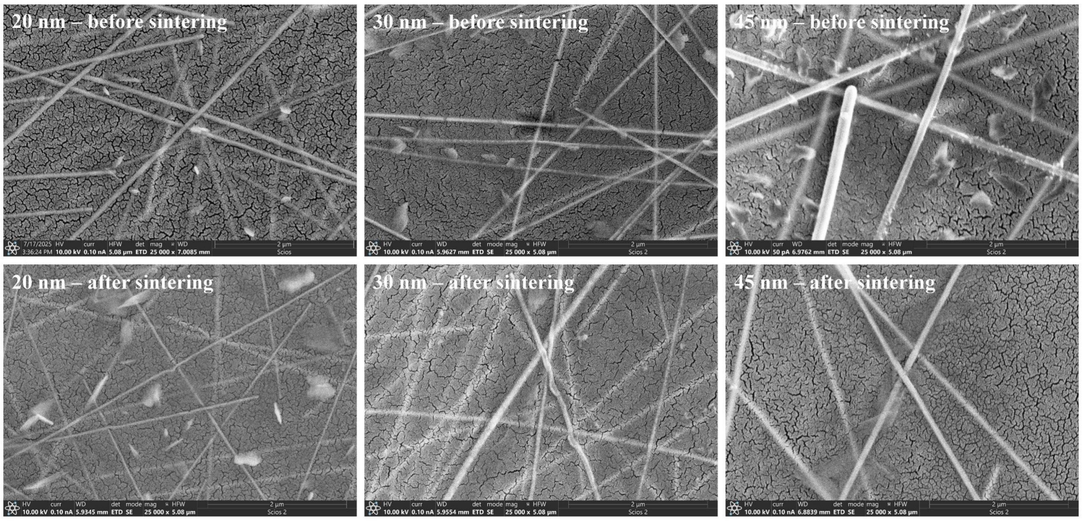

Figure S6. SEM images (Magnification 25000) of 20, 30 and 45 nm AgNW TCFs before and after sintering

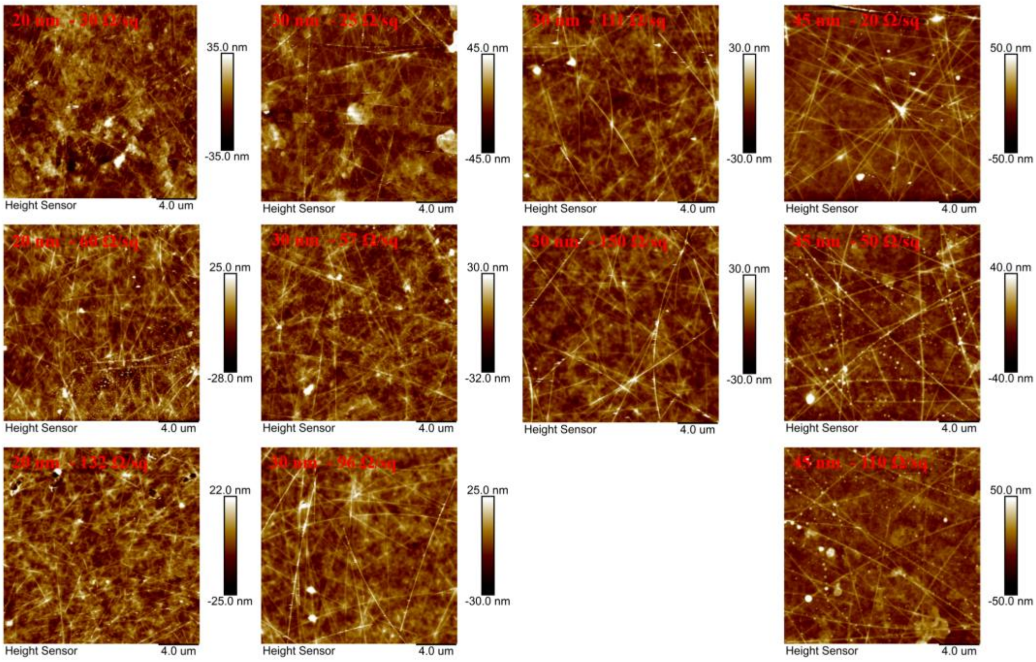

Figure S7. AFM images of 20, 30 and 45 nm AgNW TCFs with different sheet resistances
